# Determinants of Influenza Transmission in South East Asia: Insights from a Household Cohort Study in Vietnam

**DOI:** 10.1371/journal.ppat.1004310

**Published:** 2014-08-21

**Authors:** Simon Cauchemez, Neil M. Ferguson, Annette Fox, Le Quynh Mai, Le Thi Thanh, Pham Quang Thai, Dang Dinh Thoang, Tran Nhu Duong, Le Nguyen Minh Hoa, Nguyen Tran Hien, Peter Horby

**Affiliations:** 1 Mathematical Modelling of Infectious Diseases Unit, Institut Pasteur, Paris, France; 2 MRC Centre for Outbreak Analysis and Modelling, Department of Infectious Disease Epidemiology, Imperial College London, London, United Kingdom; 3 Oxford University Clinical Research Unit - Wellcome Trust Major Overseas Programme, Hanoi, Vietnam; 4 Department of Microbiology and Immunology, University of Melbourne, Melbourne, Australia; 5 National Institute of Hygiene and Epidemiology, Hanoi, Vietnam; 6 Ha Nam Centre for Preventive Medicine, Ha Nam, Vietnam; Plymouth University, United Kingdom

## Abstract

To guide control policies, it is important that the determinants of influenza transmission are fully characterized. Such assessment is complex because the risk of influenza infection is multifaceted and depends both on immunity acquired naturally or via vaccination and on the individual level of exposure to influenza in the community or in the household. Here, we analyse a large household cohort study conducted in 2007–2010 in Vietnam using innovative statistical methods to ascertain in an integrative framework the relative contribution of variables that influence the transmission of seasonal (H1N1, H3N2, B) and pandemic H1N1pdm09 influenza. Influenza infection was diagnosed by haemagglutination-inhibition (HI) antibody assay of paired serum samples. We used a Bayesian data augmentation Markov chain Monte Carlo strategy based on digraphs to reconstruct unobserved chains of transmission in households and estimate transmission parameters. The probability of transmission from an infected individual to another household member was 8% (95% CI, 6%, 10%) on average, and varied with pre-season titers, age and household size. Within households of size 3, the probability of transmission from an infected member to a child with low pre-season HI antibody titers was 27% (95% CI 21%–35%). High pre-season HI titers were protective against infection, with a reduction in the hazard of infection of 59% (95% CI, 44%–71%) and 87% (95% CI, 70%–96%) for intermediate (1∶20–1∶40) and high (≥1∶80) HI titers, respectively. Even after correcting for pre-season HI titers, adults had half the infection risk of children. Twenty six percent (95% CI: 21%, 30%) of infections may be attributed to household transmission. Our results highlight the importance of integrated analysis by influenza sub-type, age and pre-season HI titers in order to infer influenza transmission risks in and outside of the household.

## Author Summary

Influenza causes an estimated three to five million severe illnesses worldwide each year. In order to guide control policies it is important to determine the key risk factors for transmission. This is often done by studying transmission in households but in the past, analysis of such data has suffered from important simplifying assumptions (for example not being able to account for the effect of biological markers of protection like pre-season antibody titers). We have developed new statistical methods that address these limitations and applied them to a large household cohort study conducted in 2007–2010 in Vietnam. By comparing a large range of model variants, we have obtained unique insights into the patterns and determinants of transmission of seasonal (H1N1, H3N2, B) and pandemic H1N1pdm09 influenza in South East Asia. This includes estimating the proportion of cases attributed to household transmission, the average household transmission probability, the protection afforded by pre-season HI titers, and the effect of age on infection risk after correcting for pre-season HI titers.

## Introduction

Three to five millions severe illnesses and 250,000 to 500,000 deaths worldwide are due to the influenza virus each year [1]. To guide control policies, it is important that the determinants of influenza transmission are fully characterized. Such assessment is complex because the risk of influenza infection is multifaceted. For each individual, it depends on immunity that was acquired naturally or via vaccination; but also on the level of exposure to influenza the individual has in the community or in the household, which may vary by season, household and individual. Here, from the analysis of original data and relying on new and innovative statistical methods, we ascertain in a unifying and integrative framework the relative contribution of variables that influence these different mechanisms.

This task is challenging because both protection and exposure are imperfectly characterized; and uncertainties about one may affect estimates for the other. For example, for haemagglutination-inhibition (HI) assays which are extensively used in the approval process for influenza vaccines [2], [3], it is generally accepted that a HI titer of 1∶40 is associated with a 50% reduction in the risk of infection [4], [5]. However, it has long been acknowledged that HI titers are only an imperfect correlate of protection. For example, in 2009, the proportion of elderly people estimated to be protected against H1N1pdm09 influenza was much higher than had been suggested by pre-pandemic HI titers [6]. In the first study that characterized the protective effect of HI titers, Hobson et al [4] used a challenge design to ensure all subjects in the study had the same level of exposure to influenza; but such approach is expensive and can only be applied to healthy adults. In non-experimental settings, however, it is harder to control for heterogeneity in individual exposures to influenza due to the difficulty to track down all potential sources of infection.

Case-ascertained household transmission studies have been extensively used to quantify exposure in the household setting [7]–[13]. In this design, community-based influenza cases, that are labelled as index cases, are recruited via primary care practices or outpatient clinics. Symptoms of the index case and their household members are then monitored for one to two weeks following symptoms onset in the index case; virological samples may also be collected. However, since the follow-up of each household starts with an influenza case, this approach cannot be used to reliably quantify exposure from the community or to estimate the relative contributions of households and the community in the general epidemic. Furthermore, as index cases must have sufficiently severe symptoms to make contact with a healthcare provider and then have sufficiently high viral loads to be detected by laboratory tests for influenza, there may be a selection bias towards more infectious cases, which may lead the probability of transmission in the household to be overestimated.

An alternative, less common design offers a more representative view of the role of households in influenza transmission. It is based on a cohort of households that are recruited prior to an epidemic and followed up during the epidemic [14], [15]. Although the timing and source of infection is typically unobserved, collection of serum samples at baseline and after the epidemic makes it possible to determine serologically which subjects were infected. Statistical methods exist to estimate from such data the probability of transmission from other household members and from the community [16]–[18]. However, they become cumbersome and numerically intractable as the number of categories of individuals (e.g. child/adult or low/intermediate/high HI titers) or the size of the social unit of interest (e.g. here households) increase [17], [19]. As a consequence, to our knowledge, it has never been possible to evaluate the protective effect associated with HI titers in such a framework, preventing a more integrated analysis of the determinants of influenza transmission.

Here, from the analysis of the large Ha Nam household cohort study [20] conducted from 2007 to 2010 in Vietnam and relying on new and innovative statistical methods [19], we ascertain in a unifying and integrative framework the protective effects associated with HI titers and age, along with the relative contributions of households and the community in influenza transmission. Differences by subtype are also investigated. The analysis makes it possible to ascertain potential biases in case-ascertained household transmission studies which are extensively used for early assessment at the start of influenza pandemics [8], [10]–[12]. The analysis also documents influenza household transmission in South East Asia, which has received somewhat less attention than in Western countries [9], [15], [21]–[23].

## Materials and Methods

### Data

Samples were collected from a household-based cohort of 940 participants in 270 households in a single community in semi-rural northern Vietnam as previously described [20]. None of the participants had ever received influenza immunisation. Participants aged 5 years or older were asked to provide serial blood samples at times when national influenza surveillance data indicated that influenza circulation was minimal. The samples described here were collected over a period of three consecutive influenza seasons, from December 2007 through April 2010. Serological samples were collected between 1st–7th December 2007 (bleed 1), 9th–15th December 2008 (bleed 2), 2nd–4th June 2009 (bleed 3), and on the 3rd April 2010 (bleed 4). This provided three sets of paired samples either side of an influenza transmission season: 548 paired samples for season 1 (2008), 501 paired samples for season 2 (Spring 2009), and 540 paired samples for season 3 (Autumn 2009). In season 1, the influenza A virus strains detected in the cohort through ILI surveillance were A/H1N1/Brisbane/59/2007-like and A/H3N2/Brisbane/10/2007-like; in season 2, they were A/H1N1/Brisbane/59/2007-like and A/H3N2/Perth/16/2009-like; and in season 3, it was A/H1N1/California/7/2009-like. There was co-circulation of influenza B Yamagata lineage and Victoria lineage in both season 1 and season 2, with a predominance of Yamagata lineage in season 1 and Victoria lineage in season 2.

For each season and subtype, analysis was restricted to households with at most 1 individual for whom paired serum samples were missing.

### Laboratory methods

Influenza hemagglutination inhibition (HI) assays were performed according to standard protocols [WHO 2011 manual]. The seasonal influenza A viruses used were isolated from participants' swabs or from swabs taken from patients presenting in Ha Noi in the same season and propagated in embryonated hen's eggs or in MDCK cells (ATCC). A reference antigen supplied by WHO (A/H1N1/California/7/2009-like) was used to assess season 3/pandemic sera. A single influenza B virus isolated from a participant during 2008 was used to assess serum for both the first and second seasons. The virus had a titer of 320 with B/Wisconsin/1/2010 (Yamagata) reference antisera and of <10 with B/Brisbane/60/2008 (Victoria) antisera. Each virus was first assessed for haemagglutination of erythrocytes from chickens, guinea pigs and turkeys then titrated with optimal erythrocytes. Serum was treated with receptor destroying enzyme (Denka Seiken, Japan) then heat inactivated and adsorbed against packed erythrocytes. Eight 2-fold dilutions of serum were made starting from 1∶10 and incubated with 4 HA units/25 µl of virus. Appropriate erythrocytes were added and plates read when control cells had settled. Virus, serum and positive controls were included in each assay. Pre- and post-season sera were tested in pairs. Each serum was tested in a single dilution series. The HI titer was read as the reciprocal of the highest serum dilution causing complete inhibition of RBC agglutination, partial agglutination was not scored as inhibition of agglutination. If there was no inhibition of HI at the highest serum concentration (1∶10 dilution) the titer was designated as 5.

### Case definition

Influenza virus infection was defined as a ≥4-fold increase in antibody titer from pre-season to post-season titers, with post season titers ≥40. For the purposes of analysis low, intermediate, and high pre-season HI titers were defined as ≤1∶10, 1∶20–1∶40, and ≥1∶80 respectively.

### Notations

Data were collected for 3 different seasons *s* = 1…3: 2008 (*s* = 1), Spring 2009 (*s* = 2) and Autumn 2009 (*s* = 3). We classify the influenza virus into 4 different categories *v* = 1…4: seasonal A(H1N1) (*v* = 1); seasonal A(H1N1) (*v* = 2); seasonal B (*v* = 3); pandemic A(H1N1) (*v* = 4). A set of *k* = 1…*K* households are under study. Household *k* ( = 1…*K*) is of size *n_k_*. Individuals are categorized in two types: children i.e. aged ≥5 to ≤15 y.o. and adults.

### Transmission model

A subject may be infected by influenza subtype *v* in the community (i.e. outside the household) or by another household member. Here, we define a generic model for the occurrence of these events.

During season *s*, the probability that subject *i* from household *k* has contacts in the community that would lead to infection by influenza subtype *v* is defined as 
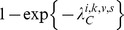
. The force of infection from the community 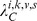 is modelled as:
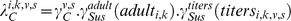
where 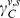 measures the force of infection for subtype *v* during season *s*, 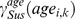 captures the susceptibility of adults relative to children (i.e children are the reference group) and 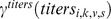 captures the effect of pre-season titers, with 3 categories low (≤10), intermediate (20–40) and high (≥80) (reference category: ≤10).

The probability that subject *i* gets infected if household member *j* is infected is defined as 
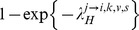
 with
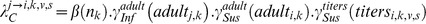
where 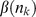 measures the transmission rate as a function of household size *n_k_* (the rate can be inversely proportional to *n_k_* [7] or independent of *n_k_*, depending on model variant), 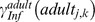 captures the infectivity of adults relative to children (i.e. children are the reference group).

### Inference

**Figure 1:**
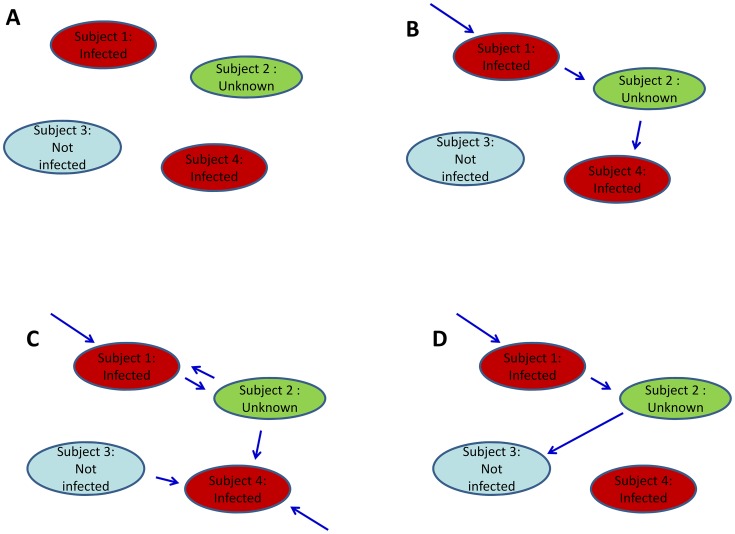
Final size data and methods to estimate transmission parameters. **A.** Example of final size data for a household of size 4. Subjects 1 and 4 were infected; subject 3 was not; diagnostic for subject 2 was missing. **B**. Example of digraph consistent with the final size data. For inference, data are augmented with a digraph (blue arrows) that informs on the transmission process. If there is an edge from the community to subject *i*, subject *i* was infected (this is the case for subject 1). If there is an edge from subject *j* to subject *i*, it means that if subject *j* was infected then subject *i* was infected too. **C**. Another example of digraph consistent with the data. We note that certain digraphs may allow more than 1 possible route of transmission. For example, subject 4 could have been infected in the community or by subject 2. **D**. Example of digraph that is not consistent with the data. This is because this digraph would imply that subject 3 was infected but subject 4 was not.

It is challenging to estimate parameters of the transmission model from final size data because the chains of transmission are not observed. Here, we consider a simplified version of the approach developed by Demiris and O'Neill [19] to tackle the problem. A household of size *n* is represented by a random directed graph with *n* vertices (Figure 1). Each vertex represents a household member. Edges are added to represent the unobserved chain of transmission. Two types of edges are possible. If there is an edge between subject *j* and subject *i*, it means that subject *i* is infected if subject *j* gets infected. If there is an edge between the community and subject *i*, it means that subject *i* gets infected.

For a given digraph, it is possible to derive the likelihood function [19]. However, since the chains of transmission are unobserved, different configurations for the edges of the digraph may be consistent with the final size data (Figure 1). The digraph is therefore considered as ‘augmented data’ [24]. The joint posterior distribution of parameters and augmented data is explored by Markov chain Monte Carlo sampling. The algorithm explores the set of digraphs consistent with the data and estimates therefore correctly capture uncertainty about the digraph (see Text S1 for technical details).

We use a Uniform prior *U([0; 10,000])* for all parameters except those characterising relative infectivity or relative susceptibility (i.e. to a reference group). For this latter class of parameters, following [8], we choose a log-Normal prior *LN(0,1)*. This prior satisfies the invariance condition that for example the ratio (adult susceptibility/child susceptibility) has the same prior as the ratio (child susceptibility/adult susceptibility). In particular, it gives equal probabilities to the relative susceptibility of children versus adults being larger or smaller than 1.

Since the households under study represent only a fraction of households in the study area [20], we assume here that households are independent of each other. The assumption of independence, which is standard in this type of analysis [8], [14], [16], [25], [26], substantially reduces the computational burden compared with that of the more general model of Demeris et al [19].

A simulation study was carried out to investigate the performances of the statistical approach.

### Model comparison

Once the model structure has been defined and methods to estimate the parameters of the model from that data are available, different model variants may be considered. For example, the effect of pre-season HI titers may be the same for all subtypes, may vary by subtype, by age group etc… Here we consider a large number of possible model variants. Each of them is fitted to the data and we determine the model variant that provides the best fit to the data. This model comparison exercise is essential to better characterize key dependencies in household transmission. We use the Deviance Information Criterion (DIC) for model comparison [27]. The smaller the DIC, the better the model. A DIC difference of 5 is considered to be a substantial improvement.

For each variable of interest, we explore the following variants:

- Effect of pre-season HI titers on susceptibility: i) no effect; ii) one threshold value (i.e. intermediate and high HI titers have a different effect than low titers); iii) two threshold values (i.e. low, intermediate and high HI titers each lead to a different protective effect). We also consider model variants in which the effect of pre-season HI titers varies with age.
- Effect of age on susceptibility: i) no effect; ii) age effect similar for all subtypes; iii) age effect different for seasonal and pandemic influenza; iv) age effect different for each subtype.
- Effect of age on infectivity: i) no effect; ii) age effect similar for all subtypes.
- Effect of household size on the household transmission rate *β*: i) *β* is independent of the household size; ii) *β* is inversely proportional to the household size. We also consider models in which *β* varies with the subtype.
- Risk of community infection of children with low pre-season HI titers: i) constant; ii) varies by season; iii) varies by subtype; iv) varies by season and subtype.

In general, no satisfying version of the criterion exists for data augmentation frameworks such as the one used here [28]. This is because the likelihood of the observed data is not available. To solve this problem, we use importance sampling [29] to estimate the likelihood of the observed data and be able to derive the DIC. The likelihood is derived as follows. For each household, we simulate *N* = 2,000 epidemics in the household. The contribution of a household to the likelihood is then equal to the proportion of simulations where simulated infection statuses in the household perfectly match the observed ones (to avoid computational issues of likelihoods equal to zero, we assume that the sensitivity *Se* and specificity *Sp* of the diagnostic is not perfect, i.e. *Se* = 0.999 and *Sp* = 0.999).

### Estimating the proportion of cases attributed to household transmission and the average household transmission probability

In order to estimate the proportion of influenza cases that may be attributed to household transmission, we simulate epidemics in households where i) all parameters are drawn from the posterior distribution and ii) all parameters are drawn from the posterior distribution except the within household transmission rate which is assumed to be null. The case counts difference between i) and ii) gives the proportion of cases that may be attributed to household transmission.

For each pair of case-household contact in the dataset, we calculate the associated probability of transmission under the assumption that the case was the first or the only infected in the pair and derive the average household transmission probability across all pairs.

### Model adequacy

We compare the observed final size distribution with the one simulated with parameters drawn from the posterior distribution.

### Ethics statement

The research was approved by the institutional review board of the National Institute of Hygiene and Epidemiology, Vietnam; the Oxford Tropical Research Ethics Committee, University of Oxford, UK; and the Ethics Committee of the London School of Hygiene and Tropical Medicine, UK. All participants provided written informed consent.

## Results

Between 140 and 155 households (439–502 subjects including 95–121 children and 344–393 adults) were eligible for analysis, depending on the season and subtype. The average household size was 2.9.

**Figure 2:**
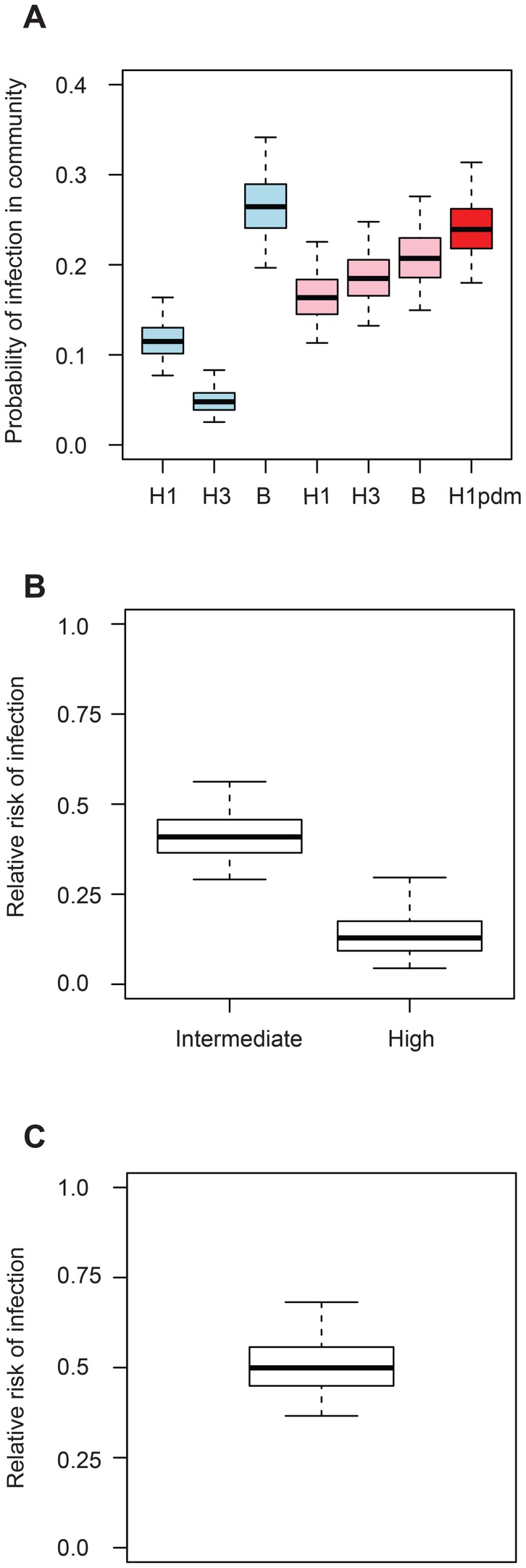
Determinants of influenza transmission in and out of the household. **A.** Probability of influenza infection from the community for children with low pre-season titres. The season is indicated by the color (blue: 2008; pink: Spring 2009; red: Autumn 2009). **B**. Relative risk of infection for intermediate (1∶20–1∶40) and high (≥1∶80) pre-season HI titres relative to low (≤1∶10) pre-season HI titres (in the household and in the community). **C**. Relative risk of infection of adults relative to children after correcting for pre-season HI titres (in the household and in the community).

Of all the model variants explored in our extensive model comparison exercise, Figure 2 summaries the characteristics of the model that had the best fit based on the DIC. The best fitting model had the following properties. The community risk of infection of children with low pre-season titers varied both with the subtype and the season (Figure 2A). It was minimum for H3N2 in 2008 and maximum for A(H1N1)pdm09 in Autumn 2009. The DIC substantially worsened if the community risk of infection of children varied with the subtype but was assumed to constant from one season to the next (ΔDIC = 24.7).

We found that high pre-season titers were protective against infection, with a reduction in the hazard of infection of 59% (95% CI, 44%–71%) for intermediate titers (20–40) and 87% (95% CI, 70%–96%) for high titers (≥80) (Figure 2B). DIC substantially worsened if the number of titer categories was reduced to 2 (ΔDIC = 20.8) or if pre-season titers were not accounted for (ΔDIC = 44.0).

Even after correcting for pre-season titers, we found that adults had half the risk of acquiring infection in the household compared to children (reduction in the hazard of infection of adults relative to children: 50%; 95% CI 32%–63%) (Figure 2C). Adding an age effect for each subtype did not improve the fit (ΔDIC = 0.2). Distinguishing pandemic versus seasonal influenza only provided a marginal improvement to the DIC (ΔDIC = −4.0) (reduction in the hazard of infection of adults relative to children for seasonal influenza: 41%, 95% CI 15%–58%; reduction in the hazard of infection of adults relative to children for pandemic influenza: 68%, 95% CI 42%–82%). Assuming the effect of age varied by subtype did not improve the fit (Figure S1; ΔDIC = 0.2). Ignoring the effect of the age of the subject on the risk of infection substantially worsened the fit (ΔDIC = 37.7). Assuming that infectivity changed with the age of the case did not improve the fit (ΔDIC = 13.2). Assuming the effect of pre-season HI titers could change with age, we found that a rise in HI titers had a slightly more pronounced effect on children than on adults (Figure S2). However, the fit of this model was not as good as that of our best fitting model (ΔDIC = 6.9).

The probability of transmission from an infected individual to another household member was 8% (95% CI, 6%, 10%) on average, and varied with pre-season titer, age and household size. In a households of size 3, the probability of transmission from an infected individual to a child with low, intermediate and high pre-season titers was estimated to be 27% (95% CI 21%–35%), 12% (95% CI, 8%, 17%) and 4% (95% CI, 1%, 9%), respectively. These probabilities dropped to 15% (95% CI 9%–23%), 6% (95% CI 4%–11%) and 2% (95% CI 0–5%), respectively, if the recipient was an adult. As has been found in studies of households in Western developed countries [7], [8], the best fitting model assumed that household transmission hazard decreased with increasing household size. Ignoring this dependency worsened the fit substantially (ΔDIC = 40.7). After correcting for these variables, estimating an effect of subtype on the probability of transmission in the household did not improve the fit (ΔDIC = 13.1). We estimated that 26% (95% CI: 21%, 30%) of cases may be attributed to household transmission. Figure S3 shows the prevalence of infection along with the estimated contribution of household transmission by season and subtype (NB: Figure 2A captures only partially variations in the prevalence of infection as the distribution of pre-season HI titers vary for each season and subtype and by age group).

The fit of the model to the data was adequate (Table 1).

**Table 1:** Observed and expected final size distribution in households with completed diagnostics.

		Number of influenza infections					Number of influenza infections					Number of influenza infections				
		0	1	2	3	4	0	1	2	3	4	0	1	2	3	4
		**H1N1, 2008**					**H3N2, 2008**					**B, 2008**				
**Household size**	**1**	13-12.3[10,13]	0-0.7[0,3]	0-0[0,0]	0-0[0,0]	0-0[0,0]	13-12.7[11,13]	0-0.3[0,2]	0-0[0,0]	0-0[0,0]	0-0[0,0]	11-11.6[9,13]	2-1.4[0,4]	0-0[0,0]	0-0[0,0]	0-0[0,0]
	**2**	14-14.1[11,16]	2-1.6[0,4]	0-0.2[0,1]	0-0[0,0]	0-0[0,0]	16-15.3[13,16]	0-0.7[0,3]	0-0[0,1]	0-0[0,0]	0-0[0,0]	13-12.7[9,15]	3-2.7[0,6]	0-0.6[0,2]	0-0[0,0]	0-0[0,0]
	**3**	14-13[9.2,15]	2-2.3[0,6]	0-0.6[0,2]	0-0.1[0,1]	0-0[0,0]	15-14.9[13,16]	1-1[0,3]	0-0.1[0,1]	0-0[0,0]	0-0[0,0]	10-11.1[8,14]	5-3[0,7]	0-0.8[0,3]	0-0.1[0,1]	0-0[0,0]
	**4**	13-14.2[10,18]	4-3.5[1,8]	2-1[0,3]	0-0.2[0,1]	0-0[0,0]	18-18.3[15,20]	1-1.4[0,4]	1-0.2[0,2]	0-0[0,1]	0-0[0,0]	13-11.9[8,16]	6-4.7[1,8.8]	0-1.7[0,4.8]	0-0.5[0,2]	0-0.1[0,1]
	**5**	7-5.6[3,8]	1-1.6[0,4]	0-0.6[0,2]	0-0.2[0,1]	0-0[0,0.8]	8-7.2[5,8]	0-0.7[0,2]	0-0.1[0,1]	0-0[0,0]	0-0[0,0]	5-3.9[1,7]	1-2.5[0,5]	2-1.1[0,3]	0-0.4[0,2]	0-0.1[0,1]
		**H1N1, Spring 2009**					**H3N2, Spring 2009**					**B, Spring 2009**				
**Household size**	**1**	11-12[10,13]	2-1[0,3]	0-0[0,0]	0-0[0,0]	0-0[0,0]	12-11.7[10,13]	1-1.3[0,3]	0-0[0,0]	0-0[0,0]	0-0[0,0]	13-11.9[10,13]	0-1.1[0,3]	0-0[0,0]	0-0[0,0]	0-0[0,0]
	**2**	10-10.1[8,12]	2-1.7[0,4]	0-0.3[0,2]	0-0[0,0]	0-0[0,0]	7-9.8[7,12]	4-1.9[0,5]	1-0.3[0,2]	0-0[0,0]	0-0[0,0]	9-9.6[6,12]	2-2[0,5]	1-0.4[0,2]	0-0[0,0]	0-0[0,0]
	**3**	10-8.2[5,11]	0-2[0,5]	1-0.7[0,2]	0-0.1[0,1]	0-0[0,0]	6-7.7[4.2,10]	5-2.3[0,5]	0-0.8[0,3]	0-0.2[0,1]	0-0[0,0]	6-7.9[5,10]	4-2.2[0,5]	1-0.8[0,3]	0-0.1[0,1]	0-0[0,0]
	**4**	12-10.3[7,14]	2-3.2[0,7]	1-1.1[0,3]	0-0.3[0,2]	0-0[0,1]	9-9.9[6,13]	4-3.5[0,7]	1-1.2[0,4]	1-0.3[0,2]	0-0.1[0,1]	10-9.5[6,13]	4-3.6[1,7]	1-1.4[0,4]	0-0.4[0,2]	0-0.1[0,1]
	**5**	4-4[2,7]	3-1.9[0,4]	0-0.8[0,3]	0-0.3[0,1]	0-0.1[0,1]	5-4.3[2,7]	2-1.8[0,4]	0-0.7[0,2]	0-0.2[0,1]	0-0.1[0,1]	6-4[2,6]	1-2[0,4]	0-0.7[0,2]	0-0.3[0,1.8]	0-0.1[0,1]
		**H1N1pdm, Autumn 2009**														
**Household size**	**1**	14-12.3[10,14]	0-1.7[0,4]	0-0[0,0]	0-0[0,0]	0-0[0,0]										
	**2**	7-9.1[6,12]	5-2.3[0,5]	0-0.6[0,2]	0-0[0,0]	0-0[0,0]										
	**3**	11-12.1[8,16]	7-4.9[1.2,9]	2-2.3[0,5]	0-0.7[0,3]	0-0[0,0]										
	**4**	10-7.8[4,12]	3-4.5[1,8]	2-2.3[0,5]	0-1.1[0,3]	1-0.3[0,1.8]										
	**5**	1-2.6[0,5]	3-1.7[0,4]	2-1[0,3]	0-0.5[0,2]	0-0.2[0,1]										

Each element of the table has the format ‘observed frequency – expected (posterior mean) frequency [95% Credible Interval]’.

In a simulation study we found all parameters could be estimated from the data and no important systematic bias was detected (Table S1). Out of 10 simulated datasets and 11 parameters, there was 94% probability that the simulation value was in the 95% CI.

## Discussion

We have characterised the determinants of transmission of seasonal (H1N1, H3N2, B) and pandemic H1N1pdm09 influenza from a household cohort study conducted in 2007–2010 in Vietnam.

We estimated that the household Secondary Infection Risk (proportion of household contacts infected by an index case, SIR) was approximately 8% on average. This is broadly consistent with estimates of SAR derived from case-ascertained studies, when diagnosis of contact cases is based on RT-PCR laboratory confirmation (median SIR_PCR_: 8%; range: 3%, 38%; *n* = 12) or on a clinical case definition of Febrile Acute Respiratory Illness (median SAR_FARI_: 11%; range: 3%, 37%; *n* = 18) [12]. Lau et al [12] also reported two estimates of the proportion of household contacts who seroconverted of 20% [30] and 27% [31]. As expected, these proportions are larger than 8% since they capture transmission from the index case but also from the community for the whole duration of the epidemic. The similarity between our estimates and those derived from case-ascertained studies validates the use of case-ascertained studies as a way to obtain representative estimates of influenza household transmission. Overall, we estimated that 26% (95% CI: 21%, 30%) of influenza infections may be attributed to household transmission. This is consistent with other estimates in the literature [32].

We also estimated the risk factors for household transmission and the risk of infection. Pre-season titer and age had a strong impact on the risk of infection. An HI titer of 40 is generally accepted to give a 50% reduction in the risk of infection [5]. Here we found a slightly more subtle effect of pre-season titer, with the risk of infection decreasing incrementally with HI titer and the reduction being as high as 90% for HI titer ≥80. Even after correcting for pre-season titers, we found that adults had half the risk of acquiring infection compared to children. This supports the idea that HI titer is an imperfect correlate of protection. There is growing evidence that antibodies directed at the stalk domain of the HA protein may be important mediators of protection that accumulates with repeated exposure to influenza viruses but which is not detectable by the HI assay [33]. Consistent with other studies [7], [8], [34], we found that the household person-to-person transmission probability decreased with increasing household size.

Ours is the only contemporary study to prospectively assess the transmission of influenza in a random selection of all households (including those without children) in an unimmunised community over multiple seasons. The use of a final-size model based on serology minimizes the under-ascertainment inherent in studies that detect only symptomatic cases. As such we believe these results are the best available assessment of the risk of acquisition of influenza in the household and the community.

The earlier analysis of this dataset [20] simply reported empirical infection rates by age based on a four-fold or greater increase in HI titers between paired sera, and did not estimate any other transmission parameters nor influences on the probability of transmission. The analysis presented in this manuscript therefore adds substantial new insights including estimates of the probability of transmission from an infected individual to another household member, the proportion of infections acquired in the household and the community, and how the probability of infection is affected by pre-season HI titers, age and household size

This study has some limitations. First, the HI assay has imperfect sensitivity and specificity [35], [36]. As a consequence, the infection status of some individuals may be incorrectly classified. The use of microneutralization assay to detect pH1N1 seroconversions would have increased the sensitivity. The average number of households per season was relatively small (about 150). However, the study was run over 3 seasons and looked at multiple different subtypes (H1N1, H3N2, B, H1N1pdm09), for a total of 6 pairs season/subtype. This means that the amount of information contained in these data is roughly that of a study of 6×150 = 900 households run over 1 season and for 1 subtype. This explains why the credible intervals for most parameters are relatively narrow. We were unable to assess transmission risks in children aged less than 5 years, since serum samples were not obtained from these subjects.

Here, we disentangled the relative contributions of households and the community in the risk of influenza infection. This was made under the assumption that households were independent of each other and that all individuals of an age group were exposed to the same risk of infection in the community. Although standard in such analyses [8], [14], [16], [25], [26], in practice, the risk of infection in the community may have a spatial component, potentially leading to higher transmission rates between households that are close to each other. However, we were unable to test this assumption here since our dataset was not spatially structured. Estimating the effect of space on influenza transmission will be an important step forward. This can for example be done from the analysis of household serological cohort studies in which the spatial location of each household is be documented [37]. Ideally, one would like to integrate such analysis in the framework of Demiris and O'Neill [19], so that the contributions of households and space can be characterized in a single and coherent framework. This is an important subject for future research.

This study considerably extends previously limited evidence on influenza transmission in non-Western countries. It also validates the use of case-ascertained studies as a way to obtain representative estimates of influenza household transmission. This has important implications for early assessment of household transmission in future pandemics, as case-ascertained studies are the only household design that can be used close to real-time.

## Supporting Information

Figure S1 **Relative risk of infection of adults relative to children for the different subtypes, after correcting for pre-season HI titres.**
(PDF)

Figure S2 **Comparison of the risk of infection of children and adults with low (L), intermediate (I) and high (H) HI titres in two different models.** The best fitting model (blue) assumes that the effect of pre-season HI titres is similar across age groups while the alternative model (pink) assumes it may vary by age group. Children with low pre-season HI titres correspond to the reference group.
(PDF)

Figure S3 **Proportion of subjects infected and contribution of households.** **A–B**: Proportion of adults (A) and of children (B) infected for each season and subtype. The black bar indicates the proportion of subjects estimated to be infected in the household. **C–D**: Proportion of adult (C) and child (D) cases estimated to be infected in the household. These proportions are given for H1N1, H3N2 and B in 2008 and Spring 2009 (2009 Spr) and for H1N1pdm09 in Autumn 2009.
(PDF)

Table S1 **Simulation study to investigate the performance of the statistical approach.** Ten datasets with a structure similar to that of the original data were simulated with parameter values equal to their posterior mean in the best fitting model. Each dataset was analyzed with our approach. For each parameter, the table gives the simulation value, the mean of point estimates, the average length of 95% CI, the number of times the simulation value is in the 95% CI.
(PDF)

Text S1 **Description of the MCMC algorithm.**
(PDF)
