# Supplementary figures and images for: Determinants of Influenza Transmission in South East Asia: Insights from a Household Cohort Study in Vietnam
Source: PLoS Pathog. 2014 Aug 21;10(8):e1004310. doi: 10.1371/journal.ppat.1004310 (PMC4140851; doi:10.1371/journal.ppat.1004310)

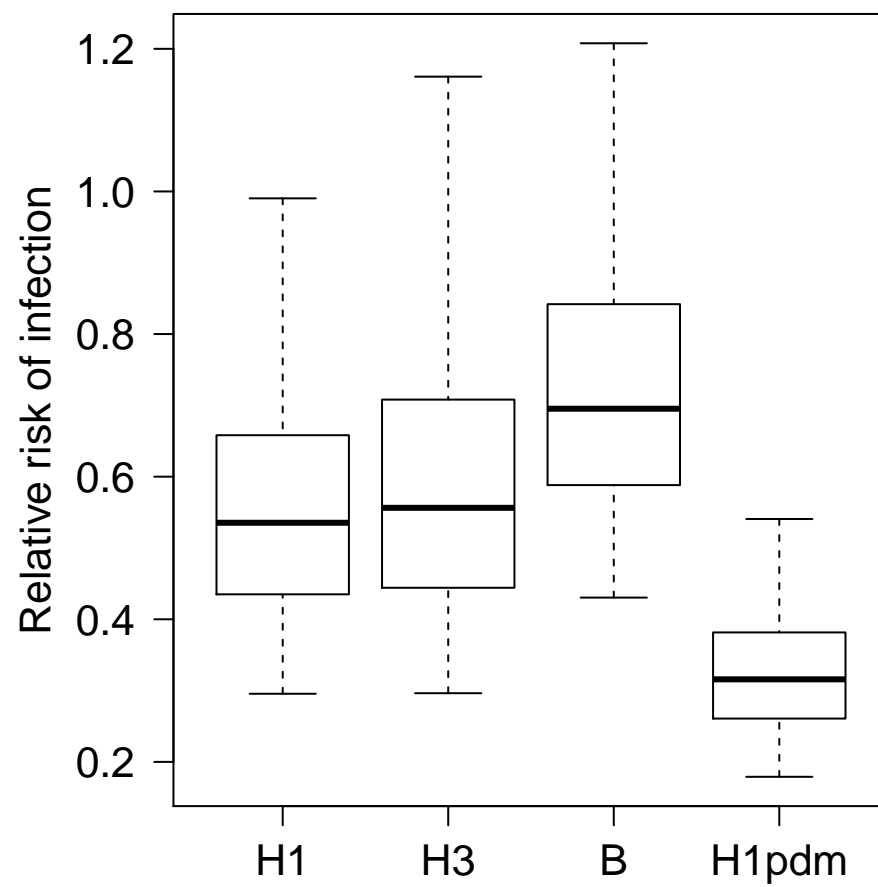

Supplement: Figure S1 — Relative risk of infection of adults relative to children for the different subtypes, after correcting for pre-season HI titres. (PDF) [file ppat.1004310.s001.pdf]

**Children**

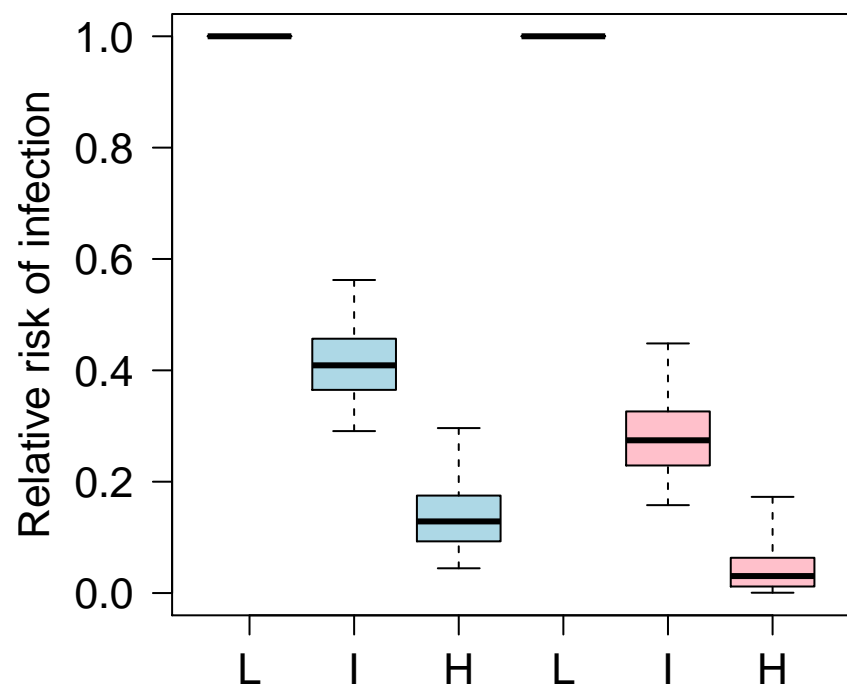

**Adults**

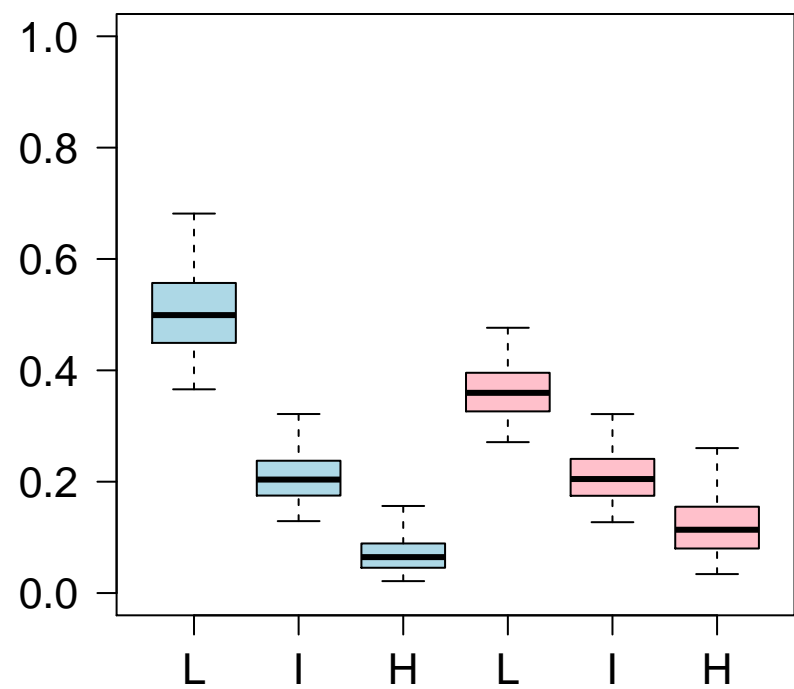

Supplement: Figure S2 — Comparison of the risk of infection of children and adults with low (L), intermediate (I) and high (H) HI titres in two different models. The best fitting model (blue) assumes that the effect of pre-season HI titres is similar across age groups while the alternative model (pink) assumes it may vary by age group. Children with low pre-season HI titres correspond to the reference group. (PDF) [file ppat.1004310.s002.pdf]

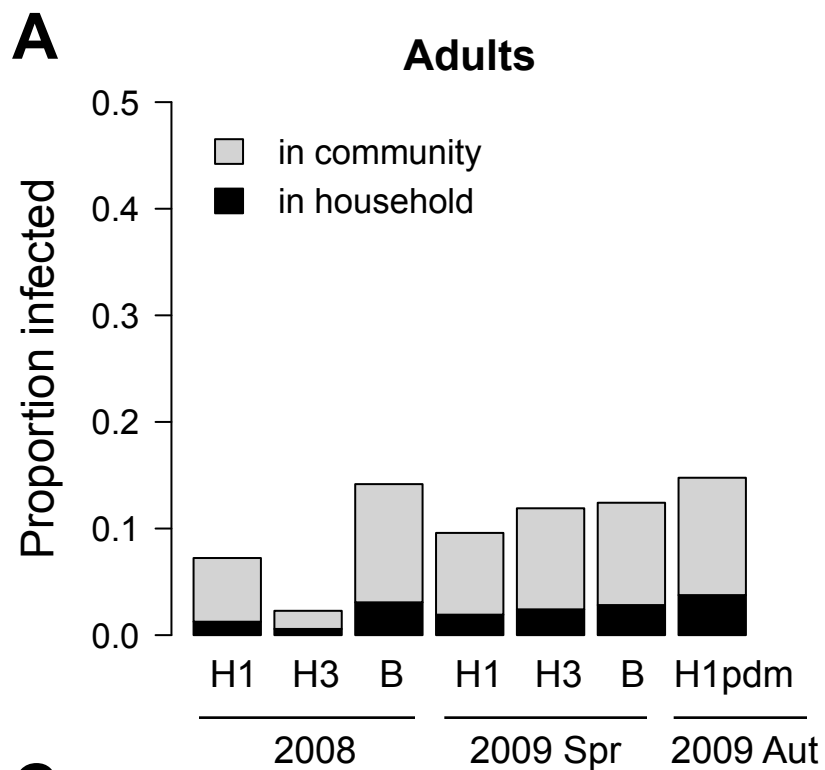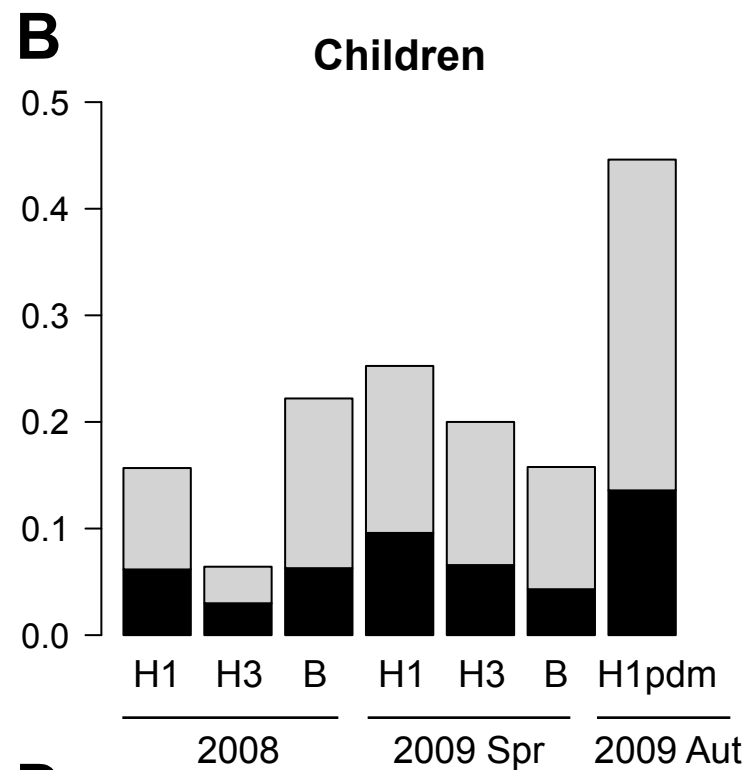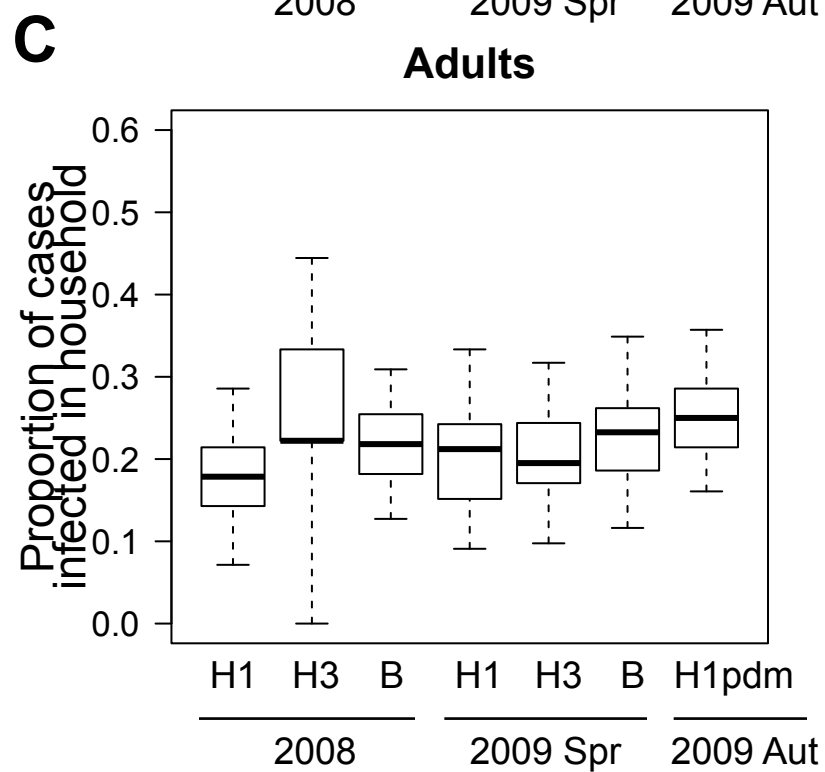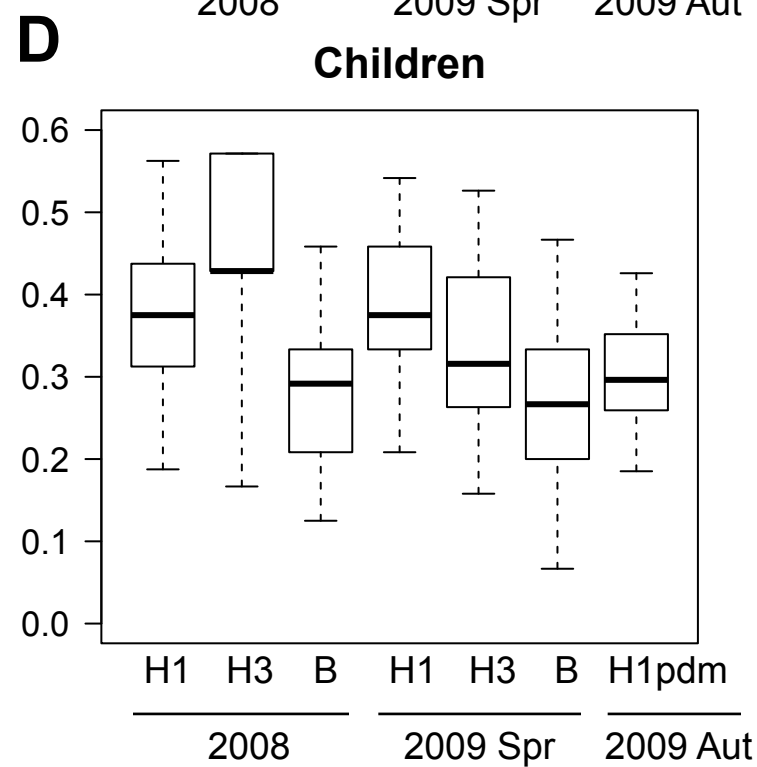

Supplement: Figure S3 — Proportion of subjects infected and contribution of households. A–B: Proportion of adults (A) and of children (B) infected for each season and subtype. The black bar indicates the proportion of subjects estimated to be infected in the household. C–D: Proportion of adult (C) and child (D) cases estimated to be infected in the household. These proportions are given for H1N1, H3N2 and B in 2008 and Spring 2009 (2009 Spr) and for H1N1pdm09 in Autumn 2009. (PDF) [file ppat.1004310.s003.pdf]
