# Supplementary material for: Determinants of Influenza Transmission in South East Asia: Insights from a Household Cohort Study in Vietnam
Source: PLoS Pathog. 2014 Aug 21;10(8):e1004310. doi: 10.1371/journal.ppat.1004310 (PMC4140851; doi:10.1371/journal.ppat.1004310)
Supplement: Table S1 — Simulation study to investigate the performance of the statistical approach. Ten datasets with a structure similar to that of the original data were simulated with parameter values equal to their posterior mean in the best fitting model. Each dataset was analyzed with our approach. For each parameter, the table gives the simulation value, the mean of point estimates, the average length of 95% CI, the number of times the simulation value is in the 95% CI. (PDF) [file ppat.1004310.s004.pdf]

Table S1: Simulation study to investigate the performance of the statistical approach. Ten datasets with a structure similar to that of the original data were simulated with parameter values equal to their posterior mean in the best fitting model. Each dataset was analyzed with our approach. For each parameter, the table gives the simulation value, the mean of point estimates, the average length of 95% CI, the number of times the simulation value is in the 95% CI.

Parameter		Simulation Value	Mean	Average length of 95% CI	Number of times simulation values in 95% CI (out of 10 datasets)
Relative risk of infection of adults		0.5	0.59	0.39	10
Risk of infection in community for children with low titres	H1N1 - 2008	0.12	0.11	0.09	10
	H3N2 - 2008	0.05	0.04	0.05	10
	B - 2008	0.31	0.27	0.19	9
	H1N1 - Spring 2009	0.18	0.16	0.12	10
	H3N2 - Spring 2009	0.2	0.2	0.14	10
	B - Spring 2009	0.23	0.2	0.15	8
	H1N1pdm - Autumn 2009	0.27	0.27	0.17	9
Household transmission		0.32	0.33	0.19	10
Relative risk of infection	Intermediate titres	0.41	0.34	0.24	8
	High titres	0.13	0.16	0.28	10
