# Supplementary material for: Determinants of Influenza Transmission in South East Asia: Insights from a Household Cohort Study in Vietnam
Source: PLoS Pathog. 2014 Aug 21;10(8):e1004310. doi: 10.1371/journal.ppat.1004310 (PMC4140851; doi:10.1371/journal.ppat.1004310)
Supplement: Text S1 — Description of the MCMC algorithm. (PDF) [file ppat.1004310.s005.pdf]

**Text S1: MCMC algorithm to explore the joint posterior distribution of parameters and augmented digraphs**

We developed an estimation algorithm based on that of Demeris and O'Neill [1]. More details about the approach, its philosophy and implementation can be found in [1].

# 1 Random digraphs

To model transmission in a household of size N, we consider a random directed graph (digraph) on *N* vertices labelled 1…*N* (for each individual of the household), plus an extra vertice labeled *C* that represents the community. The probability to add an edge from household member *j* to household member *i* is given in equation (2) in the main manuscript. The probability to add an edge from the community *C* to household member *j* is given in equation (1) in the main manuscript. The probability to add an edge from any household member to the community is null.

Presence of an edge from the community *C* to subject *i* means that subject *i* is infected. Presence of an edge from subject *j* to subject *i* means that subject *i* is infected if subject *j* is infected.

# 2 Final outcome data and augmented digraph

Final outcome data for the household consists of a vector $\{y_1,\ldots y_N\}$ where $y_i$=1 if subject *i* was infected with influenza during the season; $y_i$=0 if he/she wasn't; and $y_i$=*NA* if infection status is unknown.

We are going to augment the data with a random digraph that is consistent with the final outcome data. This augmented digraph is represented with a matrix *G* made of *N* rows and *N+1* columns. This matrix is made of 0s and 1s. An example of such a matrix for a household of size 3 is as follows:

| | subject 1 | subject 2 | subject 3 | Community C |
|---|---|---|---|---|
| subject 1 | 0 | 0 | 0 | 1 |
| subject 2 | 1 | 0 | 0 | 0 |
| subject 3 | 0 | 1 | 0 | 0 |

The matrix G is interpreted as follows.

- If *G[i,Community]*=1, individual *i* is infected;
- If *G[i,j]*=1, individual *i* is infected if individual *j* is infected.

With these rules, it is straightforward to derive, for a given matrix *G*, the associated vector of final outcomes for household members *x(G)*. For example, for the digraph presented in the example above, all household members were infected.

# 3 Hierarchical structure of the model

Denote θ the parameters of the model. The joint distribution of parameters and the augmented digraph is as follows:

$$P(y,G,\theta)=P(y\,|\,G)P(G\,|\,\theta)P(\theta)$$

where the first, second and third terms correspond to the observation model, the transmission model and the prior model respectively.

The observation model ensures that the augmented digraph *G* is consistent with the data *y*:

$$P(y\,|\,G)=\prod_{i:y_i\in\{0,1\}} \mathrm{I}\{y_i=x_i(G)\}$$

where $I\{u\}=1$ if $u=1$ and $I\{u\}=0$ otherwise.

The transmission and the prior models are described in the methods section of the main text.

# 4 MCMC algorithm

We developed an MCMC algorithm to explore the joint posterior distribution of parameters and the augmented digraph.

Parameters were updated independently on the log-scale with a standard Metropolis Hastings algorithm. The variance of the proposal was tuned so that the acceptance rate was around 20%.

In practice, as explained in Demiris and O'Neill, exploration of the augmented digraph *G* can be restricted to the subset of potential cases, i.e. individuals who are or who might be cases (i.e. $y_i$=1 or $y_i$=NA). This is because: (i) if there is an edge from a case to a non-case, the augmented digraph will be inconsistent with the data (and therefore be rejected); (ii) modelling edges from non-cases does not provide any information. This substantially reduces the dimension of augmented digraphs that need to be explored.

Assume that in the household, there are *n* potential cases, made of $n_1$ cases (i.e. $y_i$=1) and $n_{NA}$ individuals without diagnoses (i.e. $y_i$=*NA*). We use the following independence sampler to update the digraph:

- For an individual *i* who was diagnosed as a case ($y_i$=1):

- Draw the number $x$ of edges leading to subject $i$ uniformly in 1, …, $n$. Note that there are $n$ edges leading to subject $i$ _ $n$-1 from other potential cases and 1 from the community.
- Uniformly draw the $x$ edges among the $n$ possible edges.

- For an individual $i$ who did not have a diagnosis ($y_i$=$NA$):
    - Same as for those with a positive diagnosis except that this time, the number of edges is uniformly drawn in 0,…, $n$.

The acceptance rate for this step is 26%.

# 5 References


1. Demiris N, O'Neill PD (2005) Bayesian inference for stochastic multitype epidemics in structured populations via random graphs. Journal of the royal Statistical Society Series B 67: 731-745.
